# First-in-man mesenchymal stem cells for radiation-induced xerostomia (MESRIX): study protocol for a randomized controlled trial

**DOI:** 10.1186/s13063-017-1856-0

**Published:** 2017-03-07

**Authors:** Christian Grønhøj, David H. Jensen, Peter V. Glovinski, Siri Beier Jensen, Allan Bardow, Roberto S. Oliveri, Lena Specht, Carsten Thomsen, Sune Darkner, Katalin Kiss, Anne Fischer-Nielsen, Christian von Buchwald

**Affiliations:** 1 Department of Otorhinolaryngology, Head and Neck Surgery and Audiology, Rigshospitalet, University of Copenhagen, Section 2071, Blegdamsvej 9, 2100 Copenhagen, Denmark; 2 Department of Plastic Surgery, Breast Surgery and Burns, Rigshospitalet, University of Copenhagen, Blegdamsvej 9, 2100 Copenhagen, Denmark; 3 Department of Dentistry and Oral Health, Vennelyst Boulevard 9, DK-8000 Aarhus C, Denmark; 4 Department of Oral Medicine, Department of Odontology, Faculty Of Health and Medical Sciences, University of Copenhagen, Nørre Allé 20, DK-2200 Copenhagen N, Denmark; 5 Cell Therapy Facility, The Blood Bank, Department of Clinical Immunology, Rigshospitalet, University of Copenhagen, Blegdamsvej 9, 2100 Copenhagen, Denmark; 6 Department of Oncology, Rigshospitalet, University of Copenhagen, Blegdamsvej 9, 2100 Copenhagen, Denmark; 7 Department of Radiology, Rigshospitalet, University of Copenhagen, Blegdamsvej 9, 2100 Copenhagen, Denmark; 8 Department of Computer Science, University of Copenhagen, Universitetsparken 5, 2100 Copenhagen, Denmark; 9 Department of Pathology, Rigshospitalet, University of Copenhagen, Section 2071, Blegdamsvej 9, 2100 Copenhagen, Denmark

**Keywords:** Mesenchymal stem cells, Xerostomia

## Abstract

**Background:**

Salivary gland hypofunction and xerostomia are major complications following radiotherapy for head and neck cancer and may lead to debilitating oral disorders and impaired quality of life. Currently, only symptomatic treatment is available. However, mesenchymal stem cell (MSC) therapy has shown promising results in preclinical studies. Objectives are to assess safety and efficacy in a first-in-man trial on adipose-derived MSC therapy (ASC) for radiation-induced xerostomia.

**Methods:**

This is a single-center, phase I/II, randomized, placebo-controlled, double-blinded clinical trial. A total of 30 patients are randomized in a 1:1 ratio to receive ultrasound-guided, administered ASC or placebo to the submandibular glands. The primary outcome is change in unstimulated whole salivary flow rate. The secondary outcomes are safety, efficacy, change in quality of life, qualitative and quantitative measurements of saliva, as well as submandibular gland size, vascularization, fibrosis, and secretory tissue evaluation based on contrast-induced magnetic resonance imaging (MRI) and core-needle samples. The assessments are performed at baseline (1 month prior to treatment) and 1 and 4 months following investigational intervention.

**Discussion:**

The trial is the first attempt to evaluate the safety and efficacy of adipose-derived MSCs (ASCs) in patients with radiation-induced xerostomia. The results may provide evidence for the effectiveness of ASC in patients with salivary gland hypofunction and xerostomia and deliver valuable information for the design of subsequent trials.

**Trial registration:**

EudraCT, Identifier: 2014-004349-29. Registered on 1 April 2015.

ClinicalTrials.gov, Identifier: NCT02513238. First received on 2 July 2015.

The trial is prospectively registered.

**Electronic supplementary material:**

The online version of this article (doi:10.1186/s13063-017-1856-0) contains supplementary material, which is available to authorized users.

## Background

Xerostomia is the term used for a subjective feeling of dry mouth. Xerostomia can be coexisting with or without a reduced secretion of saliva, although xerostomia is generally perceived only when unstimulated whole saliva flow rate is reduced by more than 40–50% [1]. Thus, a decreased (salivary gland hypofunction) or pathologically reduced saliva secretion (hyposalivation) will severely impact quality of life and may lead to dental decay. The three main causes of severe xerostomia and hyposalivation are adverse effects of medication intake (polypharmacy and/or xerogenic medications), Sjögren’s syndrome, and radiation therapy for head and neck cancer. With regards to cancer chemotherapy, studies suggest that in some patients it may induce temporary salivary gland hypofunction and xerostomia during and following treatment, while other patients are not affected to a noticeable extent; however, no firm conclusions can be drawn from the medical literature [2]. Radiation therapy plays a major role in the curative treatment of most head and neck cancers, either as a single modality, or in combination with chemotherapy, surgery, or both. Radiation therapy significantly increases local tumor control and chance of survival, but despite more advanced methods (intensity-modulated radiation therapy, IMRT), a significant proportion of the radiation is deposited in the normal tissue surrounding the tumor. The long-term effect of radiation on salivary gland tissue is deterioration of gland function. [3, 4]. Salivary gland dysfunction after radiation therapy predisposes to a variety of undesirable conditions directly or indirectly as a result of decreased salivary flow rate as well as changed composition and increased viscosity of saliva and include xerostomia, impairment of oral functions due to insufficient wetting (i.e., speech, chewing, and swallowing) and reduced lubrication of mucosal surfaces and of the ingested food. Furthermore, the oral mucosa is prone to frictional trauma and ulceration. In addition, a reduced salivary flow rate results in a reduced clearance of the oral cavity, thus leading to microbial overgrowth which, in addition to other factors, may result in rampant dental caries, dental erosion, and oral candidiasis [2, 5].

The average total dose range which represents the threshold for a significant reduction in salivary flow rate is 26–39 Gray (Gy) [6–10]. The dose causing toxicity in 50% of individuals (TD 50) is likely to be approximately 40 Gy [11], which is similar to the TD 50 [12] estimated for submandibular hypofunction [13]. The methods used to reduce the incidence of xerostomia after radiation is prevention (including more advanced radiation techniques (IMRT or proton therapy), radio-protective agents (amifostine and Tempol [14]), and the more recently proposed stem cell-sparing radiation therapy [15]).

A number of strategies to improve salivary gland function after radiation therapy have been developed, but all are symptomatic treatments only able to stimulate the function of the residual salivary gland tissue or provide short-term lubrication. These strategies include pharmacological agents; for example, sialogogues (pilocarpine, cevimeline, etc.), saliva stimulants (sugar-free, nonerosive lozenges, chewing gum, and custom-made, nonerosive weak-acidic candy) [16], and the use of oral lubricants and saliva substitutes [17].

Stem cells have been identified as a potential treatment modality for a wide variety of cell degenerative disorders by virtue of their ability to differentiate into different specialized cell types. Most of the early work on stem cells has been performed in embryonic stem (ES) cells which are derived from the inner cell mass of a blastocyst embryo. The clinical use of these cells as therapeutic agents is currently very limited, both due to histocompatibility problems and to their potential ability to form malignant teratomas. Problems with histocompatibility have been improved with the development of induced pluripotent stem cells (iPS cells). In contrast to ES and iPS, which are both pluripotent stem cells, i.e., the ability to differentiate into cells deriving from all three primary germ layers, ectoderm, mesoderm, and endoderm; adult stem cells are defined as multipotent, i.e., with a narrower differential ability. In this respect, mesenchymal stem cells (MSCs) have gained considerable attention, since they are readily available from, e.g., bone marrow and adipose tissue, and have been characterized and investigated in a wide variety of preclinical studies and clinical trials.

Originally described more than 40 years ago, MSCs reside in almost all connective tissues [18]. More recently, there has been an increasing awareness that MSCs have a number of interesting secretory paracrine bystander characteristics including anti-inflammatory, antiapoptotic, immunomodulatory, angiogenic, and trophic (tissue-regenerating) properties. Notably, MSCs have shown promising results in preclinical studies for the treatment of xerostomia including radiation-induced xerostomia [19].

The objectives of the current trial are to assess the safety and feasibility of autologous adipose tissue-derived MSCs administered for radiation-induced salivary gland hypofunction and xerostomia in head and neck cancer patients. Head and neck cancer is the sixth most common malignancy worldwide, and the majority of these patients are at an advanced disease stage at presentation and are treated with chemotherapy, radiotherapy, or both [20, 21]. The project can potentially help to develop a clinically relevant treatment option for the growing number of patients suffering from xerostomia after radiotherapy.

## Methods

### Primary outcome

Primary outcome is change in unstimulated whole salivary flow rate.

### Secondary outcomes

Secondary outcomes are:

- Safety, including adverse events (AEs) and severe adverse events (SAEs). All measures of AEs will be graded according to Common Terminology Criteria for Adverse Events (CTCAE) [22]. Since this is a local treatment with adipose-derived MSCs (ASCs) the primary safety measures are:
  - ◦ Pain at injection site (grade 1: mild pain, grade 2: moderate pain; limiting instrumental activities of daily living (ADL), grade 3: severe pain; limiting self-care ADL)
  - ◦ Oral discomfort (grade 1: mild discomfort; not interfering with oral intake, grade 2: moderate pain; interfering with oral intake, 3: disabling pain; tube feeding or total parental nutrition indicated)
  - ◦ Infection (grade 1: localized; local intervention indicated, grade 2: oral intervention indicated (antibiotic, antifungal, antiviral), grade 3: intravenously administered (IV) antibiotic, antifungal, or antiviral agent indicated; or radiological, endoscopic, or operative intervention indicated, grade 4: life-threatening consequences; urgent intervention needed)
- Unstimulated and stimulated whole saliva and selective submandibular saliva flow rate and composition of saliva
- Complaints of xerostomia as evaluated by a physician and patient questionnaire
- Measurement of volume, fibrosis, and vascularization change of submandibular glands based on magnetic resonance imaging (MRI)
- Morphological changes of the gland tissue, e.g., degree of atrophy, fibrosis, inflammation, and amount of specialized acinic cells, including the duct/acinic/myoepithelial cell ratio, in histological sections from core-needle biopsies taken pre (baseline) and post intervention (4 months). An expert head and neck pathologist blinded to treatment of participants will evaluate the two sets of biopsies for changes

### Late complications – safety

To detect late complications or late AEs all study participants who received ASC are invited for a check-up 1 year and 3 years after treatment. Whether or not participants consent to a late check-up, the principal investigator (CG) will contact participants by telephone to ensure that no subjective complaints have befallen. In case of subjective complaints, participants will be encouraged to meet for a physical examination.

### Trial design

**Fig. 1:**
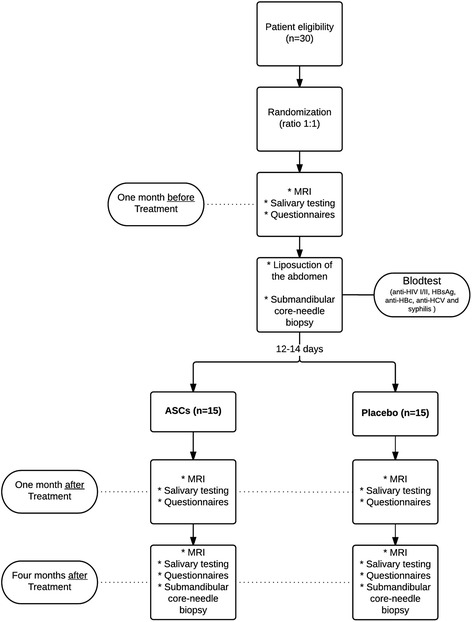
Overview of the study process

**Fig. 2:**
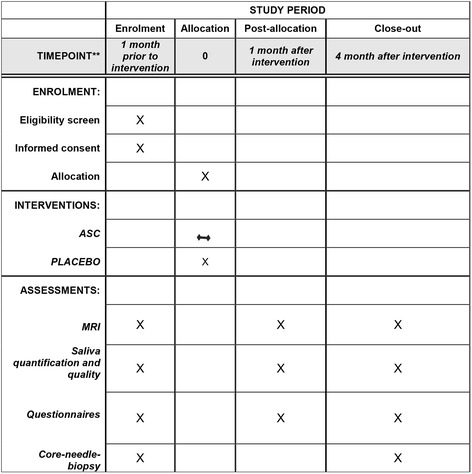
Schedule of enrollment, interventions, and assessments

This is a randomized, placebo-controlled, phase I/II trial with double blinding (see Figs. 1 and 2, and for the Standard Protocol Items: Recommendations for Interventional Trials (SPIRIT) Checklist, see Additional file 1). All participants will undergo a mini liposuction from which ASCs will be ex vivo expanded in a Good Manufacturing Practice (GMP)-approved clean room facility. The Good Clinical Practice Committee performs data monitoring, and is independent from the sponsor and has no competing interests.

One month prior to the liposuction, participants will undergo a MRI scan of the submandibular glands, fill out questionnaires on quality of life, and undergo saliva measurements. Also, all patients will have blood samples analyzed for anti-HIV I/II, HBsAg, anti-HBc, anti-HCV, and syphilis to fulfill the requirements in the Danish Tissue Act, as well as relevant kidney parameters to secure adequate renal function when using MRI contrast (see below). A core-needle sample of one of the submandibular glands and abdominal liposuction (approximately 60 ml of adipose tissue) are performed for all participants. Approximately 14 days following liposuction, participants will have ASCs or placebo injected in the submandibular glands on an outpatient basis. Subjects will subsequently undergo a saliva measurement, contrast-induced MRI and fill out questionnaires at baseline, 4 weeks post intervention and 4 months post intervention. Finally, at the 4-month visit, a small biopsy is taken from one of the two submandibular glands. The procedure takes place under local anesthesia. Histology is determined (samples are blinded to the pathologist) (Fig. 1).

### Randomization

At inclusion to the study protocol, each participant will be given consecutive numbers starting with number 1. To randomize participants to either placebo or MSCs we will use a table of random numbers, generated by a computer program (http://www.randomization.com). This table with randomization numbers will only be available to specified personnel at the Tissue Center, Department of Clinical Immunology (Rigshospitalet).

### Amount of ASCs required for injection

Based on published animal studies the amount of cells given to mice for xerostomia varies from 2 × 10^5^ to 2 × 10^6^ [23]. To convert these murine data to a realistic dose in humans we have extrapolated numbers based on the following data: the volume density of submandibular glands very closely approximates 1 mg/mm^3^ (1.06–1.07 mg/mm^3^) [24]. In the murine studies on radiotherapy, the mean gland weight was approximately 350 mg, which corresponds to an approximate volume of 350 mm^3^. Accordingly, the murine dose per gland volume is approximately 2.86 × 10^5^ to 2.86 × 10^6^ ASC/cm^3^ gland (when calculated from either 1 × 10^5^ to 1 × 10^6^). The volume of the submandibular gland in human subjects after radiotherapy is 6.6–7.9 cm^3^ [25]. This corresponds to an approximate dose per patient of between 1.9 × 10^6^ to 2.3 × 10^6^, or 1.9 × 10^7^ to 2.3 × 10^7^ cells per submandibular gland. From the above assumptions, and in order not to administer an inadequate number of cells, we have chosen to proceed with the maximum dose corresponding to 2.8 × 10^6^ ASC/cm^3^ gland, i.e., a maximum total dose per patient of approximately 4.48 × 10^7^ ASCs (see below). However, to standardize the dose administered to patients, the amount administered will be standardized to the size of their submandibular glands, as described below.

Injection of the ASCs or placebo suspensions into the submandibular glands will be performed by the principal investigator (CGL) under local anesthesia using ultrasonic guidance and sterile technique. The ASCs will be suspended in isotonic NaCl (0.9 mg/ml) and human albumin (HA) 1% to a final volume of 2 ml. Placebo will be 2 ml of isotonic NaCl (0.9 mg/ml) and HA 1%. After receiving the ASC suspension or the placebo suspension, the surgeon (the principal investigator) will identify the submandibular glands and inject the ASC suspension. Calculation of injected number of ASCs per participant rests on the following calculation:

$$ Suspensio{n}_{m l}=2.8 \times {10}^6\ \frac{ASC}{c{ m}^3} \times \kern0.5em  Volum{e}_{c{ m}^3}, $$

where volume is the volume of the submandibular gland, and a gland volume of approximately 7–8 cm^3^ is the norm. Therefore, the amount of ASCs given to each participant will be approximately:$2.8 \times {10}^6\frac{ASC}{c{m}^3} \times \kern0.5em 8 c{m}^3\times 2=4.48\ {10}^6\  ASCs$ in total.

If the expanded number of ASCs does not fulfill the above criteria, it is accepted to diminish the number to a minimum of 50% of the calculated dose.

### Justification for patient population

In order to standardize the participant population, we choose to only include participants treated for a human papilloma virus (HPV)-positive oropharyngeal squamous cell carcinoma, and to exclude patients with severe salivary gland hypofunction (hyposalivation, e.g., whole saliva flow rate <0.05 ml/min), as this population most likely will not benefit from this treatment. This will be assessed by a preliminary questionnaire and a subsequent saliva flow rate measurement before inclusion.

### Eligibility criteria

Evaluation of eligibility criteria and Consent Forms are collected by the primary investigator.

Inclusion criteria:

- Previous radiotherapy for a T1–T2 and N0, N1, or N2a, HPV-positive oropharyngeal squamous cell carcinoma with bilateral irradiation of the neck
- 2 years’ follow-up without recurrence
- Clinically reduced salivation and hyposalivation, evaluated by a screening
  - ◦ Unstimulated whole saliva flow rate in the range of 0.05–0.20 ml/min
- Informed consent
- Grades 1–3 xerostomia as evaluated by the “Udvalg for Kliniske Undersøgelser” (UKU) Side-effect Rating Scale [26]

Exclusion criteria:

- Any cancer in the previous 2 years
- Ongoing xerogenic medications
- Any other diseases of the salivary glands, e.g., Sjögren’s syndrome, sialolithiasis, etc.
- Pregnancy or planned pregnancy within the next 2 years
- Breastfeeding
- Any other disease/condition judged by the investigator to be grounds for exclusion
- Treatment with an anticoagulant that cannot be stopped during the intervention period
- Failure of expanding up to 50% of the calculated dose of ASC
- Withdrawal of informed consent

Criteria for withdrawal of subjects under study

- Pregnancy
- Infection of the transplanted site
- Allergy to local anesthetic
- Withdrawal of consent from participants
- In case of withdrawal or dropout before assessment of efficacy patients will be replaced to ensure that a total of 30 patients complete the study

### Surgical method for mini liposuction

The procedure is done in an outpatient setting, and performed under sterile conditions according to local guidelines. Six milliliters of local anesthesia (lidocaine 1%) is injected subcutaneously at two injection sites of the lateral abdomen. Two 5-mm incisions are then made and Klein’s solution is injected with a blunt infiltrator through the incisions. The fatty tissue is harvested from the abdomen with a 3-mm blunt cannula 23 cm in length, coupled to the MonoJect 60-ml syringe with a blunt and Toomey tip (Teico Healthcare). The harvested lipoaspirate is sedimented in the syringe. Any oil layer at the top or aqueous layer at the bottom is removed. By this procedure the middle layer of fat tissue is obtained and will be transported in a sterile plastic bag for the isolation of ASCs.

### Surgical method for submandibular gland biopsy

This procedure takes place in an outpatient settings. A biopsy will be taken 14 days before injecting the ASCs and 4 months after injection. The biopsy procedure is carried out under sterile conditions; in local anesthesia with lidocaine with epinephrine 0.5% an ultrasound-guided, core biopsy of one of the glands is taken. Participants are randomized for biopsy of either the right or left gland. Each biopsy will be fixed in 4% formalin and sent for histology.

### Surgical method for injection of ASCs or placebo in the submandibular gland

The procedure will be performed without local anesthesia in an outpatient setting. The surgeon will receive two syringes of the sterile ASC suspension or placebo, each of 1 ml (total of 2 ml). The suspensions are injected into each submandibular gland guided by ultrasound. The ASC suspension will be deposited in two areas (superficial and deep lobe) with equal volume per site (0.5 ml) to ensure equal distribution of the suspension. The subject will afterwards receive a small bandaid, which can be removed the same day. The participant will be administered over-the-counter pain relief.

### Assessment of xerostomia and subjective treatment outcome

The subjective effect of the treatment is assessed by a 100-mm Visual Analog Scale of xerostomia filled out by the patient [27] and a physician-rated questionnaire [28], both carried out at baseline as well as at 1 and 4 months after treatment (Fig. 1).

### Assessment of salivary flow rate and objective treatment outcome

Changes in the secretion rate of the unstimulated whole saliva in the oral cavity is probably the most important parameter for the biological development of xerostomia and accompanying pathological oral conditions. Whole saliva, e.g., the secretions from the major and minor salivary glands are mixed in the oral cavity. A precise determination of this value is crucial for the assessment of efficacy in this trial. For determination of the saliva flow rate, whole saliva will be collected between 2 p.m. and 3 p.m. for all collections to take the salivary diurnal variation into account. Participants will refrain from eating, drinking, smoking and administering oral hygiene for 2 h prior to collection. After being seated upright in a chair, subjects relax for 5 min and are then instructed to make as few movements as possible, including swallowing, during the collection. At baseline, 1 and 4 months after treatment, unstimulated whole saliva will be collected using the spitting method [29] where participants spit their saliva into a collection container over a period of 15 min. The salivary flow rate (SFR) (ml/min) is determined as the increase in weight of the container divided by the collection time in minutes. After the collection of unstimulated saliva, the subjects are instructed to chew on 1 g of sterile paraffin wax. Participants will chew for 60 s, and clear the oral cavity for saliva. Subsequently, as the glands are now in a stimulated state, participants will continue chewing the paraffin wax and expectorate into a saliva collector for the duration of 5 min. Subsequently, testing of the submandibular glands will be performed.

To assess ASC efficacy on the submandibular glands, saliva is also collected directly from the floor of the mouth in an unstimulated and stimulated state. The flow rate of the submandibular glands will be assessed by the swab method with cotton rolls placed buccally in each maxillary molar region to block the orifices of the parotid ducts and cotton rolls under the tongue in the floor of the mouth to collect submandibular/sublingual saliva. Immediately prior to the start of collection participants will be asked to swallow. Unstimulated saliva testing will be performed with neutral cotton rolls (Salivette®, Sarstedt, Nümbrecht, Germany) and stimulated saliva testing with cotton rolls with 20 mg citric acid (Salivette with citric acid®, Sarstedt, Nümbrecht, Germany). For both measurements, collections takes place during a period of 3 min. Saliva flow rates are determined by weight (1 g equals 1 ml of saliva) with cotton rolls weighed before collection and reweighed after. The flow rates are calculated as the increase in weight during collection and expressed as milliliters per minute. Saliva from the cotton rolls will be extracted by centrifugation (1500 *g*) and analyzed for its composition. From each of these collections, saliva will be aliquoted and stored at −80°C.

### Chemical analysis of saliva

Whole saliva contains a large number of bacteria and epithelial cells as well as gingival crevicular fluid. Therefore, whole saliva is normally not suited for the analysis of sensitive chemical parameters. For this purpose, noncontaminated, selectively collected saliva from individual glands: i.e., parotid and submandibular/sublingual saliva is more suitable. The following analyzes will be performed on the collected saliva: pH and bicarbonate level by ionic balance estimation [30], sodium, potassium, calcium, phosphate, chloride and fluoride [16], total protein, selected proteins [31], and amylase [32]. Chemical analysis will provide an estimate of dental and mucosal protective capacity of the saliva before and after treatment.

### MRI analysis of the submandibular glands

A 3.0-Tesla, four-channel head coil and a dual-channel neck coil are employed to obtain hsMRI images of subjects (Siemens Magnetom Verio, Erlangen, Germany).

Volumetric and tissue-specific analysis will be performed based on axial and coronal sequences with 72 slices and 4-mm slice thickness including diffusion-weighted and dynamic contrast-induced sequences. The contrast agent used is gadolinium (“Gadovist”).

### Statistical analyses

Data will be analyzed with SPSS v. 24 (IBM) or R statistics. We will use Excel or Access databases for collecting and entering data. All data will be double entered. End of trial is defined as: last patient’s last visit (LPLV).

The results on salivary flow rate will be calculated as a percentage change in salivary flow rate (from baseline) in the group of participants given ASCs compared to the score change in the control group.

Histologically, the composition of glandular tissue in the submandibular gland will be calculated from before (baseline) and 4 months after the intervention (Fig. 1). The differences between the intervention and the placebo group will be calculated by a nonpaired *t* test or, alternatively, a nonparametric test, if the conditions for parametric tests are not present. Differences are considered statistically significant if the two-sided *p* value is less than 0.05.

### Sample size calculation

The following sample size determination is based on a nonpaired *t* test. The minimal change in salivary flow rate that we want to be able to determine is 50%.

Standard deviation (SD) of a salivary flow rate measurement can, using the spitting method in healthy participants and under completely standardized conditions, be minimized to about 20%. The SD can vary greatly and we have, therefore, in this test used a value from the published medical literature [33] for persons with reduced unstimulated whole salivary flow rate (from 0.00 to 0.20 ml/min) and/or hyposalivation (<0.10 ml/min) indicating a relative SD of 58% of these groups. However, since we are interested in a comparison of change scores (change from baseline to after treatment) between the intervention and placebo groups we apply the rule of thumb that the SD of the change score equals SD/2^0.5^ which equals an SD of approximately 41%. The power is set at (1 − *β*): 90%. Significance level (*α*) is set at 0.05. This is a trial with a continuous response variable from independent control and experimental research unit with one control per experimental research unit. If the true difference in the change score between experimental and control means is 50%, we will need to study 15 participants for the experimental intervention and 15 patients as a control population in order to reject the null hypothesis that the population means of the experimental and control groups are equal with a study power of 90%. The Type I error probability associated with this test of this null hypothesis is 0.05.

All investigators will have access to the final trial dataset.

## Discussion

This first-in-man trial aims to establish the safety, efficacy, and feasibility of autologous, adipose-derived stem cell therapy for patients with radiation-induced xerostomia. In addition, it provides an opportunity to advance trial methodology for the assessment of the putative role of stem cell therapy and provide suggestions for the design of subsequent trials to verify potential efficacy. To establish the safety profile of the intervention, selection and timing of appropriate outcomes have been guided by similar intervention studies [34], and our trial design facilitates conditions for assessment of short- and long-term changes and complications.

We have strived to standardize saliva testing by performing tests at the same time of the day, and instructed and supervised by the same person (CG). All participants are fasting 2 h before the testing. This period is deemed useful to exclude any stimulants (e.g., lunch and drinking liquid) before testing but might, especially for participants with hyposalivation (e.g., participants with unstimulated whole saliva flow rate ≤0.10 ml/min), induce almost no secretion. Further, we perform in total four saliva measurements per visit (two tests for whole saliva (unstimulated/stimulated), and two tests for the submandibular glands (unstimulated/stimulated)) totaling to an effective period of 26 min of saliva testing. This is possibly too extensive for some participants meaning that the glands will, when testing the submandibular glands, not be capable of further secretion. Testing of the submandibular glands is performed following the stimulated testing of all glands (whole saliva). It might be an advantage to test the submandibular glands in a post-stimulated state, but it should be noted that the glands are stimulated. Testing of the submandibular glands is performed by placement of cotton rolls at the salivary gland orifices under the tongue, and consequently the rolls will also collect saliva from the sublingual glands.

We have adjusted numbers of cell according to gland size based on experience from preclinical studies, but whether this is transferable to humans and the dose is adequate remains unknown.

Following the liposuction 12–14 days prior to treatment, a core-needle biopsy of one of the submandibular glands (participants are randomized for biopsy of either the right or left gland) is performed. It cannot be excluded that this procedure might induce salivation and influence the effect of the ASCs. It is well-documented that ASCs secrete bioactive molecules that provide a regenerative microenvironment for a variety of injured tissues to limit damage and facilitate a regenerative response [35]. In this context, it is unknown whether injection to the submandibular glands regenerates only damaged tissue from the biopsy or also generates new or improved tissue.

In connection with the injection of the MSCs into the salivary glands, there is a minimal risk of AEs, such as pain, infection, and bleeding. The risk of these AEs is less than 0.5% using similar procedures. A theoretical risk of a possible carcinogenic effect in the treatment of MSC has been discussed due to their production of growth factors such as epidermal growth factor (EGF). These considerations are particularly relevant since MSCs are used in participants previously diagnosed with cancer. However, in model systems of cancer, the effect of MSCs on cancer growth is controversial as they have both been shown to be inhibitory and stimulatory [36, 37]. Vascular endothelial growth factor (VEGF) is a known facilitator of angiogenesis, and might be a mediator of the potential effect of ASCs [38].

Based on numerous trials including studies with local injection of MSCs with a total of more than 1000 participants no increase in the incidence of cancer is detected [39, 40]. Data on ex vivo expanded MSCs have not, despite extensive research, indicated malignant transformation [39], including a study that demonstrated that the injection of a very high dose of ASCs (240 × 10^6^ MSC/kg) revealed no signs of cancer, organ toxicity, or change in body weight in mice after 12 months [41].

It is estimated that the risks of AEs in the experiment is outweighed by the benefits of participation. If the treatment has a clinically significant effect and is safe, patients suffering from radiation-induced xerostomia could be offered a simple, minimally invasive procedure with few AEs and risks, which is in contrast to the current suboptimal treatments.

### Trial status

This is an investigator-initiated phase I/II trial funded by Candy’s Foundation. The ethics committees have given full approval. The date of first enrollment was 22 May 2015. The trial is in the recruitment phase.

## Additional file

Additional file 1: SPIRIT Checklist. (DOC 121 kb)

## Abbreviations

ASC: Adipose-derived MSC therapy
EGF: Epidermal growth factor
IMRT: Intensity-modulated radiation therapy
LPLV: Last patient’s last visit
MRI: Magnetic resonance imaging
MSC: Mesenchymal stem cell
SD: Standard deviation
VEGF: Vascular endothelial growth factor
